# Cross-shelf habitat shifts by red snapper (*Lutjanus campechanus*) in the Gulf of Mexico

**DOI:** 10.1371/journal.pone.0213506

**Published:** 2019-03-14

**Authors:** Michael A. Dance, Jay R. Rooker

**Affiliations:** 1 Department of Oceanography and Coastal Sciences, Louisiana State University, Baton Rouge, Louisiana, United States of America; 2 Department of Marine Biology, Texas A&M University (Galveston Campus), Galveston, Texas, United States of America; 3 Department of Wildlife and Fisheries Sciences, Texas A&M University, College Station, Texas, United States of America; Department of Agriculture and Water Resources, AUSTRALIA

## Abstract

Habitat shifts that occur during the life cycles of marine fishes influence population connectivity and structure. A generalized additive modeling approach was used to characterize relationships between environmental variables and the relative abundance of red snapper *Lutjanus campechanus* over unconsolidated substrate on the continental shelf (<150 m) of the U.S. Gulf of Mexico (GoM) at three different life stages: juvenile (age-0, <125 mm FL), sub-adult (age-1-2, 125–300 mm FL), and adult (age-2+, >300 mm FL). Fisheries independent data (2008–2014) were used to develop separate models for both the eastern and western GoM, and final models were used to predict the relative availability of suitable habitat for each life stage across the two regions. Predictor variables included in final models varied by age class and region, with depth, dissolved oxygen, longitude, and distance to artificial structure common to most models. Depth was among the most influential variables in all models, and preferred depth increased with increasing size/age. Regional differences in fish-habitat relationships were also observed, as relative abundance of larger red snapper over unconsolidated substrates was more closely linked to artificial structure in the eastern GoM. The location of predicted high quality habitat for juvenile red snapper was greatest on the inner Texas shelf and a smaller area east of the Mississippi River Delta, suggesting these two areas may represent important nursery grounds for the respective regions. Clear ontogenetic shifts in the spatial distribution of predicted high quality habitat were evident in both the eastern (expansion from west to east with age) and western (shift from inshore to offshore) GoM. Given the unique population dynamics between the eastern and western GoM, improving our understanding of spatial and temporal variability in habitat quality may be important to maintaining connectivity between juvenile and adult habitats, and may enhance recovery and management of red snapper stocks in the GoM.

## Introduction

Habitat use by aquatic organisms often reflects a series or suite of behavioral decisions to maximize fitness and ultimately survival to the next age class [1–3]. In the most basic sense, these decisions are based on maximizing growth while minimizing mortality [2, 4, 5]. Thus, habitat selection is inherently affected by ontogeny, as increases in body size influence both the resources an animal is able to exploit and the associated predation risk involved [1, 6, 7]. In response, multiple habitats or regions are often needed for an animal to complete its life cycle [2, 3, 8, 9], creating age-structured or size-structured populations that are spatially segregated [10]. This spatial segregation complicates our ability to identify and conserve essential habitats, as the importance of habitat patches, landscapes (seascapes), and/or regions to each life stage may vary [4, 11, 12].

Ontogenetic habitat shifts are common in marine fishes, and understanding the connectivity among habitats used by each life stage is a critical prerequisite to developing successful conservation strategies [13–16]. Reef fishes, in particular, often undergo conspicuous ontogenetic shifts across a suite of habitats (seagrass/mangrove prop roots to patch reefs to barrier reefs), often moving farther offshore (inner to outer shelf) with increasing size/age [17, 18]. The relative value of a particular area as a nursery is then dependent on the functional connectivity between essential habitats at each life stage, as areas with disconnected habitats can be less productive [11, 19]. Still, our understanding of cross-shelf connectivity in marine fishes is primarily limited to tropical coral reef-mangrove systems and estuarine dependent reef fishes [20, 21], and the degree to which similar cross-shelf habitat shifts occur in subtropical and temperate reef fishes remains poorly understood.

Here, we characterize ontogenetic shifts in habitat for a subtropical reef fish, red snapper (*Lutjanus campechanus*), within the U.S. Gulf of Mexico (hereafter referred to as GoM). Red snapper is a long-lived reef fish that inhabits subtropical shelf waters of the western Atlantic Ocean. It is arguably the most valuable reef fish in the GoM and is highly targeted by both recreational and commercial fisheries [22]. Unlike many tropical lutjanids that inhabit nearshore seagrass and/or mangrove ecosystems as juveniles, red snapper inhabit shelf habitats of low vertical complexity (sand, shell, and mud) during the juvenile life stage [23, 24]. However, similar to other reef fishes, red snapper appear to gradually move from inner to outer shelf habitats as they mature [25, 26]. Nevertheless, benthic habitat and physicochemical properties of shelf waters vary greatly across the GoM, and the relative distribution of suitable habitat across the region remains poorly documented. The aim of this study was to use a fisheries independent data set and generalized additive model framework to determine the influence of environmental factors on the distribution and abundance of red snapper over unconsolidated substrate in two different regions (eastern and western) of the GoM at three different life stages (juvenile, sub-adult, and adult). We then used these models to predict the relative availability of suitable habitat at each life stage across different regions of the GoM to characterize cross-shelf shifts in distribution that occur during ontogeny and identify regions of high habitat suitability.

## Methods

Fishery independent catch data for red snapper were obtained from trawl surveys conducted over unconsolidated substrates in shelf waters (< 150 m) of the GoM during both summer (June-July) and fall (September-October) as part of the Southeast Area Monitoring and Assessment Program (SEAMAP) from 2008 to 2014. This survey uses a stratified random sampling design to select locations based on depth and shrimp statistical zones [27]. The SEAMAP sampling protocol for trawl surveys systematically avoids untrawlable habitat (hard bottom habitats and artificial reef sites) to avoid hang ups, and selected sampling stations that occur in such habitat are relocated to the nearest trawlable location (up to 1.8 km from such structure) [27]. As a result, SEAMAP trawl surveys primarily targeted unconsolidated substrates (e.g. non-hard bottom sediments including gravel, sand, mud, coralgal, marl, and shell). A 12.2 m otter trawl was towed at each station for approximately 30 minutes [27], and the linear distance of each trawl tow was then used to calculate the area swept at each station as a measure of sampling effort. Prior to 2008, SEAMAP surveys were weighted more heavily from the Texas-Mexico border to the Florida-Alabama border, resulting in a far greater number of samples in the western versus the eastern GoM; thus, the time period of 2008–2014 was chosen for this study to better account for spatial variability in the abundance of red snapper in each region.

Red snapper captured in the surveys were divided into three age classes based on fork length (FL) to approximate age and reproductive maturity categories from previous studies [26, 28]. For the purposes of this study, individuals less than 125 mm FL were defined as juveniles (age-0), those between 125–300 mm were considered sub-adults (age 1–2), and individuals greater than 300 mm were classified as adults (age 2+). Because red snapper spawn from May to August and have a planktonic larval duration of approximately 27–30 days [23, 29], age-0 juveniles were likely not fully recruited to the trawl gear during summer surveys (June and July); therefore, we restricted analysis of this life stage to fall samples only. Although we acknowledge that trawling gear is likely more efficient at capturing juveniles and sub-adults than larger, older adults, our primary interest was to identify factors driving spatial patterns in relative abundance of red snapper within each age class. Thus, direct comparisons of catch were not made across age classes, and we assumed that spatial variability in gear efficiency was negligible.

Environmental data used for modeling were collected at each survey site at the time of sampling. Bottom temperature, dissolved oxygen, salinity, turbidity, and depth were measured at each station with a conductivity, temperature, depth profiler. Other environmental variables or metrics (sediment composition, location of natural hard bottom and artificial structure, shelf position) were extracted from archived data sources and matched to study locations or calculated in a geographic information system (ArcGIS 10.2.2). Benthic sediment data obtained from the United States Geological Survey (USGS; http://coastalmap.marine.usgs.gov/regional/contusa/GoMex/gloria/data.html) usSEABED data set [30] were kriged to create a continuous raster surface to estimate the benthic sediment composition (% rock, gravel, sand, and mud) at each survey station. Locations of standing oil and gas platforms in the GoM were obtained from the Bureau of Ocean Energy Management (BOEM; https://www.data.boem.gov/homepg/data_center/mapping/geographic_mapping.asp), and locations of artificial reefs (e.g., pre-fabricated concrete pyramids or reef balls, toppled platforms, ships) were obtained from data sets compiled by BOEM and the National Oceanic and Atmospheric Administration (NOAA; MarineCadastre.gov). These two data sets (standing platforms and artificial reefs) were combined for modeling purposes, and are collectively referred to hereafter as artificial structures. Natural hard bottom habitat was derived from coral reef habitat layers obtained from NOAA National Centers for Environmental Information (NCEI; https://www.ncddc.noaa.gov) as well as areas with sediment comprised of at least 1% rock (usSEABED) to account for hard bottom habitat that does not support coral growth. Distance to the nearest artificial structure and natural hard bottom was calculated as the shortest in-water distance from a sampling location to these features using the Cost Distance tool in the Spatial Analyst toolbox of ArcGIS 10.2.2. Distance (km) to shore and shelf edge (150-m isobath) were calculated as the linear distance from a particular station to the nearest shoreline or 150-m isobath, respectively, in ArcGIS 10.2.2. Shelf position was calculated as the distance from a station to the shoreline divided by the width of the shelf (distance from shoreline to 150-m isobath).

**Fig 1:**
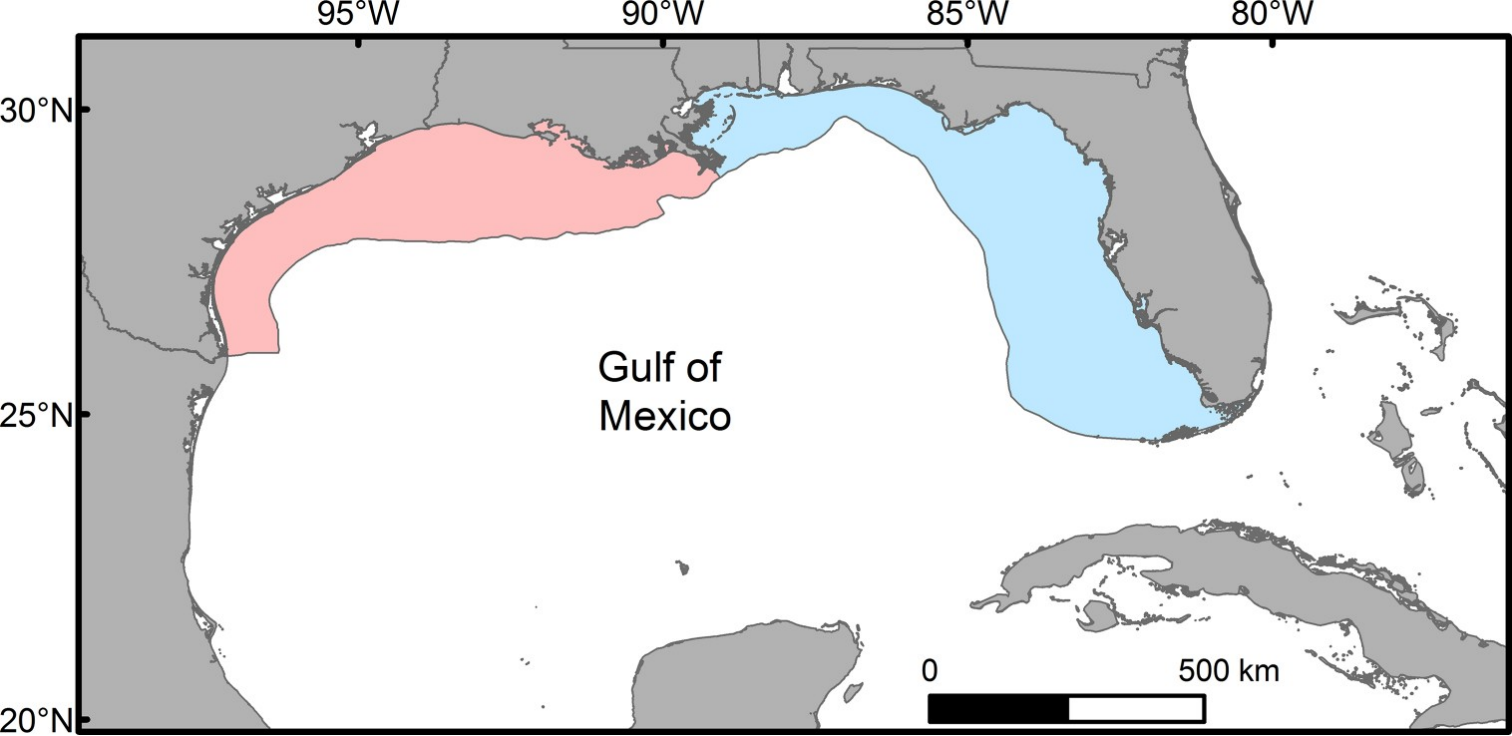
Shelf waters (< 150 m) of the U.S. Gulf of Mexico. The eastern Gulf of Mexico is shown in blue, while the western Gulf of Mexico is shown in red.

Generalized additive models (GAMs) were used to examine the influence of environmental variables on red snapper abundance over unconsolidated bottom at each age class in U.S waters of both the eastern and western GoM. Separate models were developed for each region of the GoM to account for potential differences in fish-habitat relationships between the eastern and western GoM given known demographic differences between the two regions and the fact that the regions are assessed separately in stock assessments [31, 32]. The eastern GoM was defined as shelf waters (< 150 m) of the U.S. GoM generally east of the central stem of the Mississippi River Delta (South Pass ~ 89.1° W), and the western GoM was defined as shelf waters of the U.S. GoM west of this feature (Fig 1). Red snapper catch was modeled as a count variable with effort (area swept during trawl tow) included as an offset. Models for each age class/region combination (n = 6) were fit with a negative binomial distribution using a logit link function in the *mgcv* library in R [33, 34]. Cubic regression splines were automatically penalized from a specified maximum degrees of freedom (df = 4) to prevent overfitting [35, 36] and the degree of smoothing selected by minimizing the REstricted Maximum Likelihood (REML) score [37].

A manual backwards stepwise selection procedure based on minimizing the Akaike Information Criterion (AIC) [38] and REML score was used to select predictor variables influencing red snapper catch. Approximate significance (p values) of smoothed predictor variables was used to guide the backward selection of variables; however, removal of a variable at each step in the backwards procedure was based on minimizing AIC and REML. The selection process was terminated at the step at which removal of any of the remaining variables resulted in an increase in AIC and REML. Prior to backwards selection, collinearity of predictor variables was examined using Spearman’s rank correlation coefficient (ρ) and variance inflation factor (VIF) [36, 39]. Variables with VIF > 3 were removed from analysis prior to initial model. If Spearman’s ρ > 0.7 between two variables, then each variable was tested alone in a separate GAM, and the variable that resulted in the lower AIC was entered into the initial model while the other variable was discarded. Overall model fit was also tested using the percent deviance explained (DE). Relative importance of each variable to final models was assessed by removing each variable individually from final models and examining the change in AIC (ΔAIC) and percent deviance explained (ΔDE) before and after the variable was removed [40].

Final GAMs were used to predict the relative distribution of suitable habitat for red snapper across the continental shelf of the GoM during the fall season using the predict.GAM function in R (3.3.1). Environmental data were extracted to a grid of point locations evenly spaced at a resolution of 0.2° inside the 150-m isobath. For prediction grids, bathymetry at each location was extracted from the National Geophysical Data Center (NGDC) Coastal Relief Model (3-arc-second; https://www.ngdc.noaa.gov/). To estimate bottom physicochemical conditions at each location, archived bottom temperature, DO, and salinity recorded at sampling sites during fall SEAMAP cruises were interpolated using ordinary kriging to create a continuous raster surface for each variable (0.1°), from which exact values could be extracted to each prediction point. Benthic sediment type and distance to natural and artificial structures were also extracted from raster layers used in model building. For predictions, effort at all locations was set equal to the mean area swept per trawl tow during the study period (0.03 km^2^). After all environmental values were extracted, the predict.GAM function in R (3.3.1) was used to predict the abundance of each age class of red snapper at each location. For visualization, grid points were converted to a continuous raster surface at a resolution of (0.1°), which was then smoothed using bilinear interpolation. Predicted high quality habitat was defined as the grid cells containing the upper 95% of the predicted relative abundance values for each age class across the GoM shelf. Although we expect that spatial patterns of red snapper distribution and abundance over unconsolidated substrates will be reflective of basin scale patterns of distribution for the GoM population, we chose a conservative threshold value to best account for the limitations of the SEAMAP survey in sampling natural and artificial structure. The relative distribution of predicted high quality (PHQ) red snapper habitat for each age class was then contrasted among regions in the GoM. Regions of the GoM shelf were classified into 4 coastal state groups (Texas, Louisiana, Mississippi/ Alabama, and Florida; S1 Fig) based on administrative boundaries obtained from BOEM and derived from Supreme Court fixed baselines (Texas, Louisiana, Mississippi, and Alabama) and the national baseline for Florida (2006, http://www.boem.gov/Administrative-Boundaries/). The relative proportion (based on areal coverage) of PHQ habitat occurring within each region was then calculated for each age class of red snapper and was expressed as a percentage. Finally, an index of relative habitat suitability was calculated for each region as the observed percent contribution to overall PHQ red snapper habitat divided by the expected percent contribution (based on proportion of GoM shelf within each region).

## Results

Separate models for the eastern and western GoM were developed for each age class, and deviance explained ranged from 34.7–54.3% with the highest for juvenile stage models in both regions (Table 1). Deviance explained decreased with age from juvenile (49.1%) to adult (34.7%) for eastern GoM models. In contrast, the deviance explained was 54.3% for the juvenile model in the western GoM, and the adult model (40.5%) explained a greater percent of the deviance than the sub-adult model (34.9%). Predictor variables included in final models varied widely with only depth retained in all models. However, several variables were common to at least 5 of the 6 models including dissolved oxygen, longitude, and distance to artificial structure. The relative importance of each predictor variable varied by both life stage and region, and models from each region are presented by life stage below.

**Table 1:** Environmental variables retained in final generalized additive models for juvenile (age-0), sub-adult (age-1-2), and adult (age-2+) red snapper over unconsolidated substrate of the eastern and western GoM.

**Eastern GoM**						
	**Juvenile**		**Sub-adult**		**Adult**	
**AIC = 1209.1**		**DE = 49.1**	**AIC = 2182.7**	**DE = 41.7**	**AIC = 705.2**	**DE = 34.7**
**Variable**	**Δ AIC**	**Δ DE**	**Δ AIC**	**Δ DE**	**Δ AIC**	**Δ DE**
Latitude						
Longitude	74.3	19.5	11.53	1.9	33.78	12.8
Depth	97.8	25.5	58.7	7.4	9.8	5.3
Dis. Oxy.	2.7	0.3	19.1	2.6		
Salinity			1.7	0.4		
Temperature	4.1	1.9	21.3	3.4		
Dist. Artificial	3.0	1.0	4.8	1.4	2.0	1.2
Dist. Natural					16.6	6.4
% Mud			7.8	1.8		
% Gravel						
**Western GoM**						
	**Juvenile**		**Sub-adult**		**Adult**	
**AIC = 5341.7**		**DE = 54.3**	**AIC = 7885.5**	**DE = 34.9**	**AIC = 1128.1**	**DE = 40.5**
**Variable**	**Δ AIC**	**Δ DE**	**Δ AIC**	**Δ DE**	**Δ AIC**	**Δ DE**
Latitude	116.1	6.2	187.4	7.1	16.6	3.6
Longitude	94.3	5.1	52.3	2		
Depth	88.9	5.1	122.1	4.6	96.0	16.4
Dis. Oxy.	9.2	0.7	7.6	0.9	6.1	1.4
Salinity						
Temperature	30.4	1.8	20.0	0.9		
Dist. Artificial	5.3	0.4	2.2	0.1		
Dist. Natural			24.2	1.1	3.9	1.3
% Mud			1.2	0.1		
% Gravel			4.3	0.3		

Akaike’s Information Criterion (AIC) and percent deviance explained (DE) are shown for final models. Relative importance of each predictor variable is given by the difference in AIC (ΔAIC) and DE (ΔDE) if this variable was removed from the final model. Variables include: latitude, longitude, depth, dissolved oxygen (Dis. Oxy.), salinity, temperature, distance to artificial structure (Dist. Artificial), distance to natural hard bottom habitat (Dist. Natural), percent mud, and percent gravel.

### Juvenile

**Fig 2:**
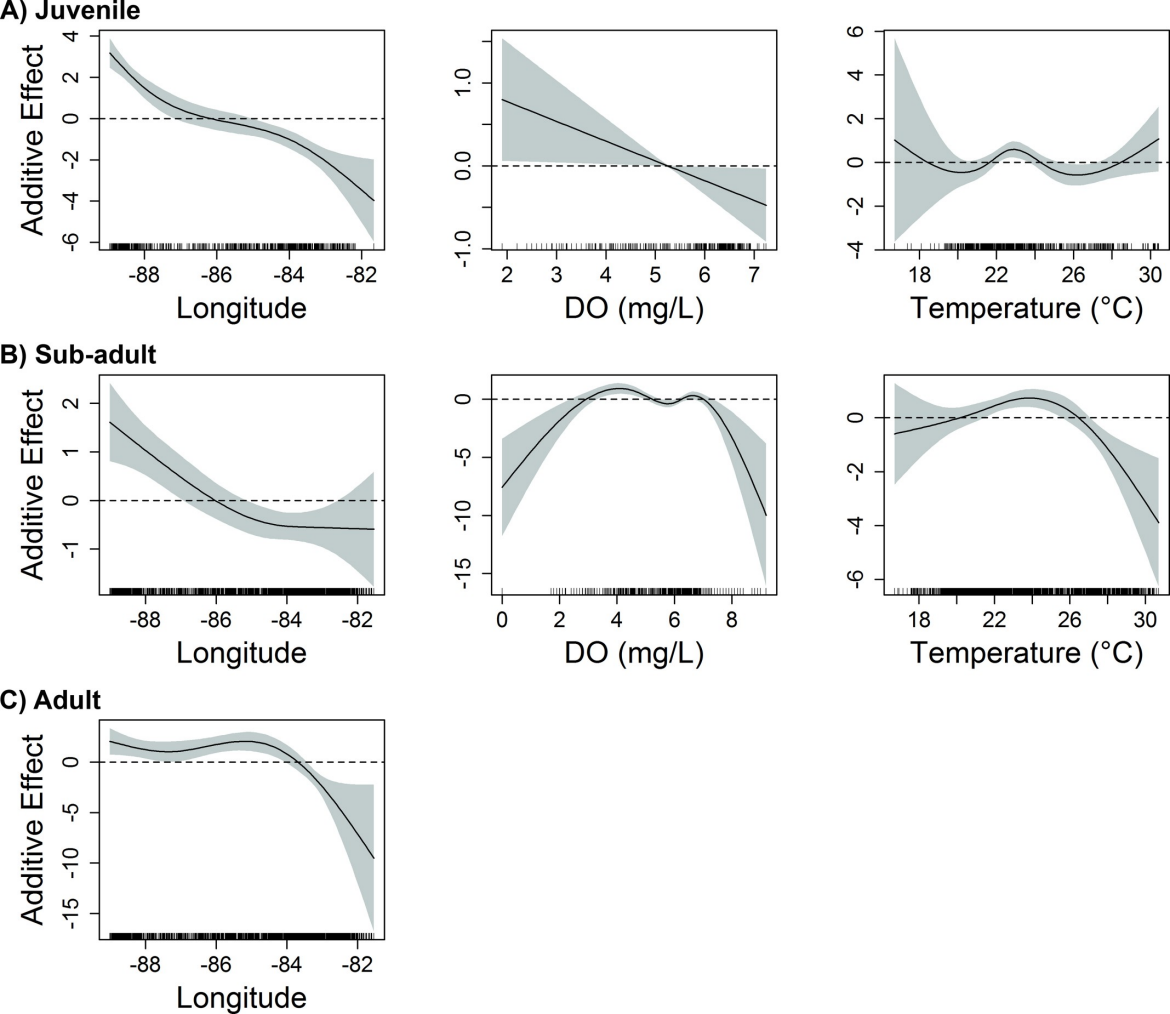
Influence of geographic and physicochemical variables on red snapper abundance in the eastern GoM. Response plots from final generalized additive models show the influence of longitude, bottom dissolved oxygen (DO), and bottom temperature on the abundance of A) juvenile (age-0), B) sub-adult (age-1-2), and C) adult (age-2+) red snapper over unconsolidated bottom in the eastern GoM. Solid lines represent smoothed values, and shaded areas represent 95% confidence intervals. Dashed lines are included at y = 0.

**Fig 3:**
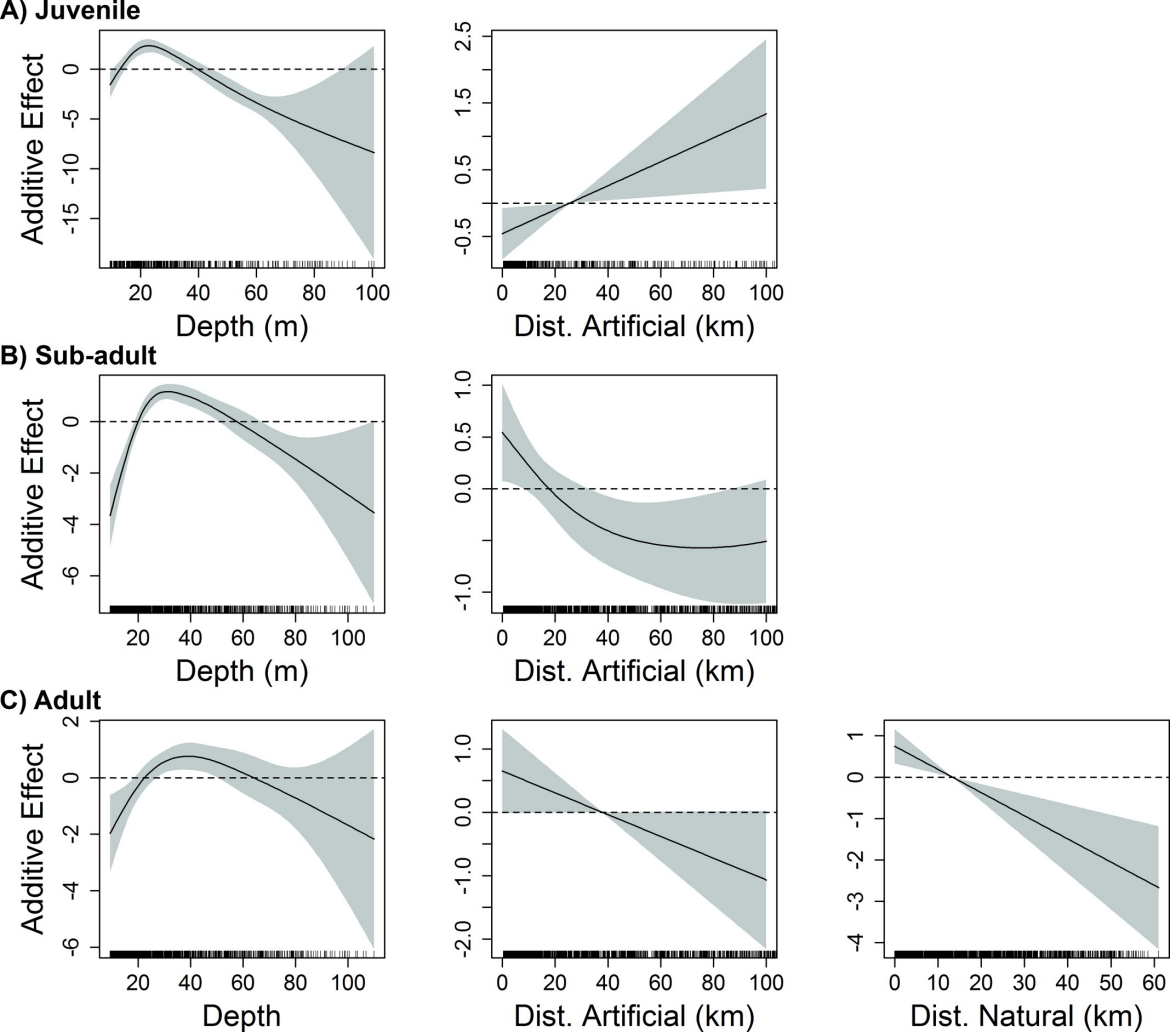
Influence of benthic habitat variables on red snapper abundance in the eastern GoM. Response plots from final generalized additive models show the influence of depth (m), distance to artificial structure (Dist. Artificial), and distance to natural hard bottom habitat (Dist. Natural) on the abundance of A) juvenile (age-0), B) sub-adult (age-1-2), and C) adult (age-2+) red snapper over unconsolidated bottom in the eastern GoM. Solid lines represent smoothed values, and shaded areas represent 95% confidence intervals. Dashed lines are included at y = 0.

Five variables were deemed influential to juvenile red snapper (age-0) abundance over unconsolidated bottom in the eastern GoM: longitude, temperature, dissolved oxygen, depth, and distance to artificial structure. The most influential variables (according to both ΔAIC and ΔDE) were depth, longitude, and temperature (Table 1). Juvenile red snapper east of the Mississippi River delta were most abundant in shallow shelf waters (depth 10–40 m) of the north-central GoM (87–89° W), and were negatively associated with proximity to artificial structure (Figs 2 and 3). Abundance decreased from west to east across the eastern GoM, and was greatest in areas of moderate to warm temperatures (21–25° C) with dissolved oxygen levels from 2–5 mg/L (Fig 2).

**Fig 4:**
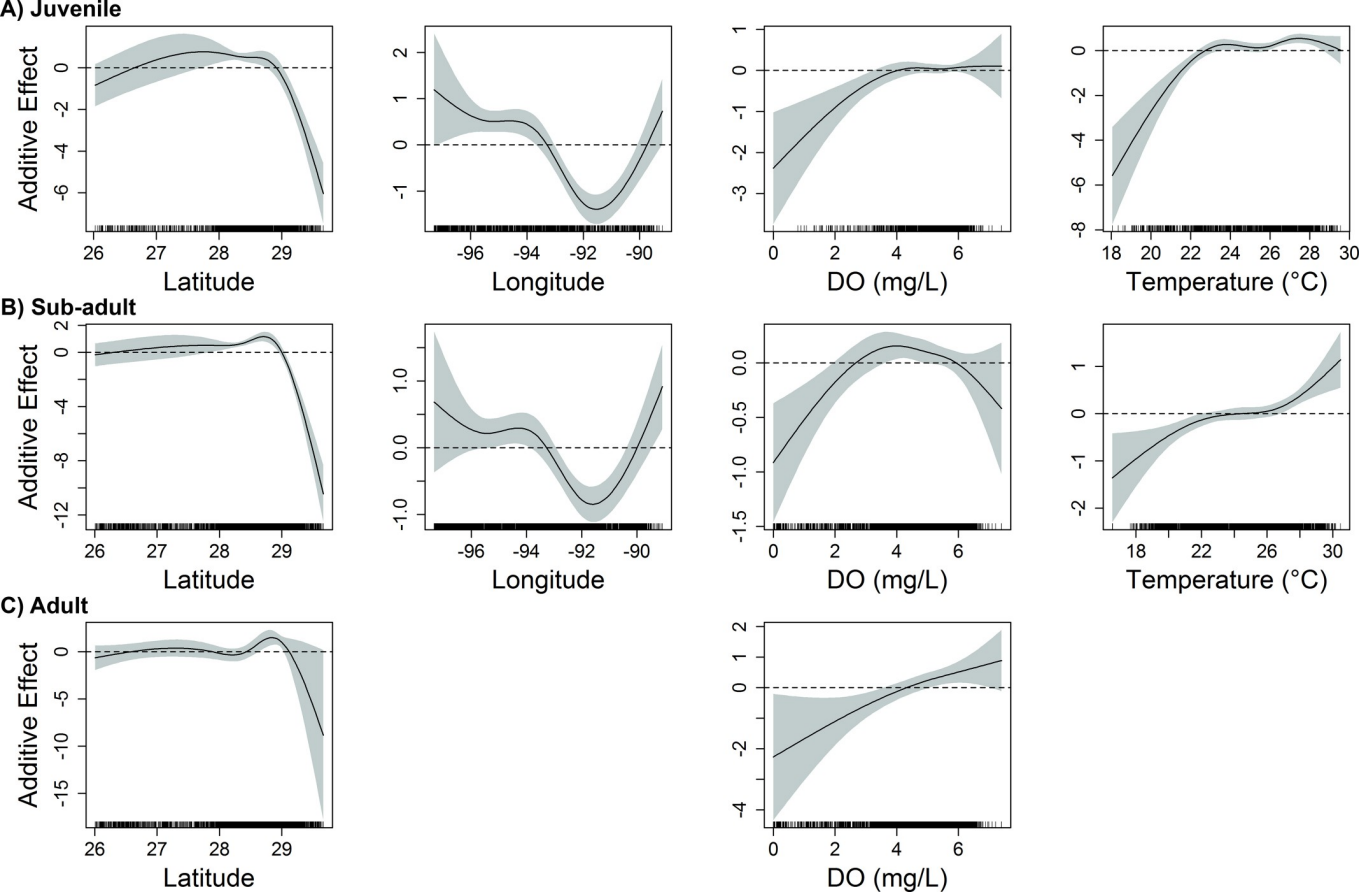
Influence of geographic and physicochemical variables on red snapper abundance in the western GoM. Response plots from final generalized additive models show the influence of latitude, longitude, bottom dissolved oxygen (DO), and bottom temperature on the abundance of A) juvenile (age-0), B) sub-adult (age-1-2), and C) adult (age-2+) red snapper over unconsolidated bottom in the western GoM. Solid lines represent smoothed values, and shaded areas represent 95% confidence intervals. Dashed lines are included at y = 0.

**Fig 5:**
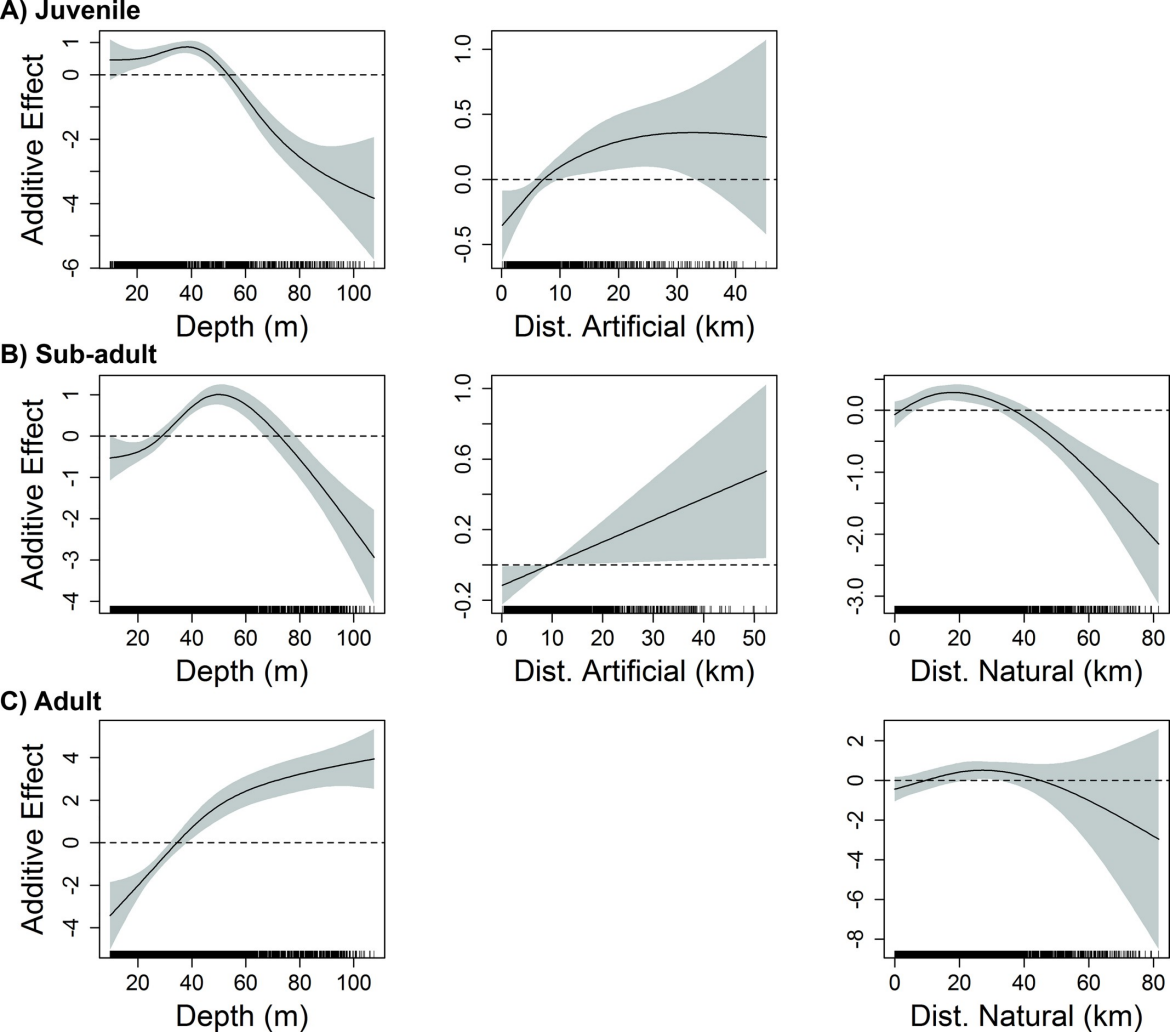
Influence of benthic habitat variables on red snapper abundance in the western GoM. Response plots from final generalized additive models show the influence of depth (m), distance to artificial structure (Dist. Artificial), and distance to natural hard bottom habitat (Dist. Natural) on the abundance of A) juvenile (age-0), B) sub-adult (age-1-2), and C) adult (age-2+) red snapper over unconsolidated bottom in the western GoM. Solid lines represent smoothed values, and shaded areas represent 95% confidence intervals. Dashed lines are included at y = 0.

Six variables were included in the final model describing variability in abundance of juvenile red snapper in the western GoM: latitude, longitude, temperature, dissolved oxygen, depth, and distance to artificial structure. The most influential variables according to ΔAIC were latitude, longitude, and depth (Table 1). The same three variables were identified as most influential to the final model based on ΔDE. Spatially, juvenile red snapper abundance was lowest at higher latitudes and generally decreased from west to east across the western GoM (Fig 4). Similar to the eastern GoM, juvenile snapper in the western GoM were most abundant over the inner shelf at depths less than 50m with moderate to warm temperatures (Figs 4 and 5). Juveniles in the western GoM were also negatively associated with artificial structures and areas of low dissolved oxygen (< 4 mg/L).

### Sub-adult

The final model for sub-adult red snapper (age 1–2) in the eastern GoM included seven variables: longitude, temperature, salinity, dissolved oxygen, depth, % mud substrate, and distance to artificial structure. The most influential variables in the model (according to both ΔAIC and ΔDE) were depth, temperature, and dissolved oxygen (Table 1). Sub-adult red snapper were most abundant at depths of 20-55m in close proximity to artificial structures (Fig 3). This life stage was also positively associated with moderate to warm temperatures (20–27° C) and dissolved oxygen from 3–7 mg/L (Fig 2). Abundance decreased substantially from west to east, with greatest abundances observed over substrates with moderate to high mud content (40–60%).

In the western GoM, nine variables were retained in the final model describing variability in abundance of sub-adult red snapper: longitude, latitude, temperature, dissolved oxygen, depth, % mud substrate, % gravel substrate, distance to natural hard bottom habitat, and distance to artificial structure. The most influential variables (according to both ΔAIC and ΔDE) were latitude, depth, longitude, and distance to natural hard bottom habitat, while the remaining five variables (temperature, dissolved oxygen, % mud substrate, % gravel substrate, and distance to artificial structure) had little influence on the ΔDE (< 1%) (Table 1). Sub-adult red snapper were most abundant at depths from 25–70 m and were generally found within a moderate distance from natural hard bottom habitat (1–30 km) (Fig 5). With the exception of a peak east of 91°W near the Mississippi River Delta, abundance generally decreased from west (97°W) to east (92°W) across much of the region, and was lowest at higher latitudes (Fig 4).

### Adult

In the eastern GoM, the final model identified four variables influencing the abundance of adult red snapper (age-2+) over unconsolidated bottom: longitude, depth, distance to artificial structure, and distance to natural hard bottom habitat. Longitude, distance to natural hard bottom habitat, and depth were the most influential variables in the final model based on ΔAIC and ΔDE (Table 1). Abundance of adult red snapper in the eastern GoM was greatest at moderate depths between 20-65m, decreasing east of 84°W (Figs 2 and 3). In addition, adult red snapper abundance increased with proximity to both artificial structure and natural hard bottom habitat (Fig 3).

Four variables were included in the final model describing variability in adult red snapper abundance in the western GoM. The most influential variable (according to both ΔAIC and ΔDE) was depth (ΔAIC = 96.0, ΔDE = 16.4%), followed by latitude, with dissolved oxygen and distance to natural hard bottom habitat also contributing (Table 1). Adult red snapper abundance was positively related to depth, with individuals rarely captured at depths less than 30 m (Fig 5). While adult red snapper were found in greatest abundance between 28.5° and 29°N, they were negatively associated with latitudes greater than 29°N (Fig 4). Adult red snapper over unconsolidated bottom were more abundant at close to moderate distances (5–40 km) from natural hard bottom habitat, and were positively associated with dissolved oxygen concentration.

### Regional contributions

**Fig 6:**
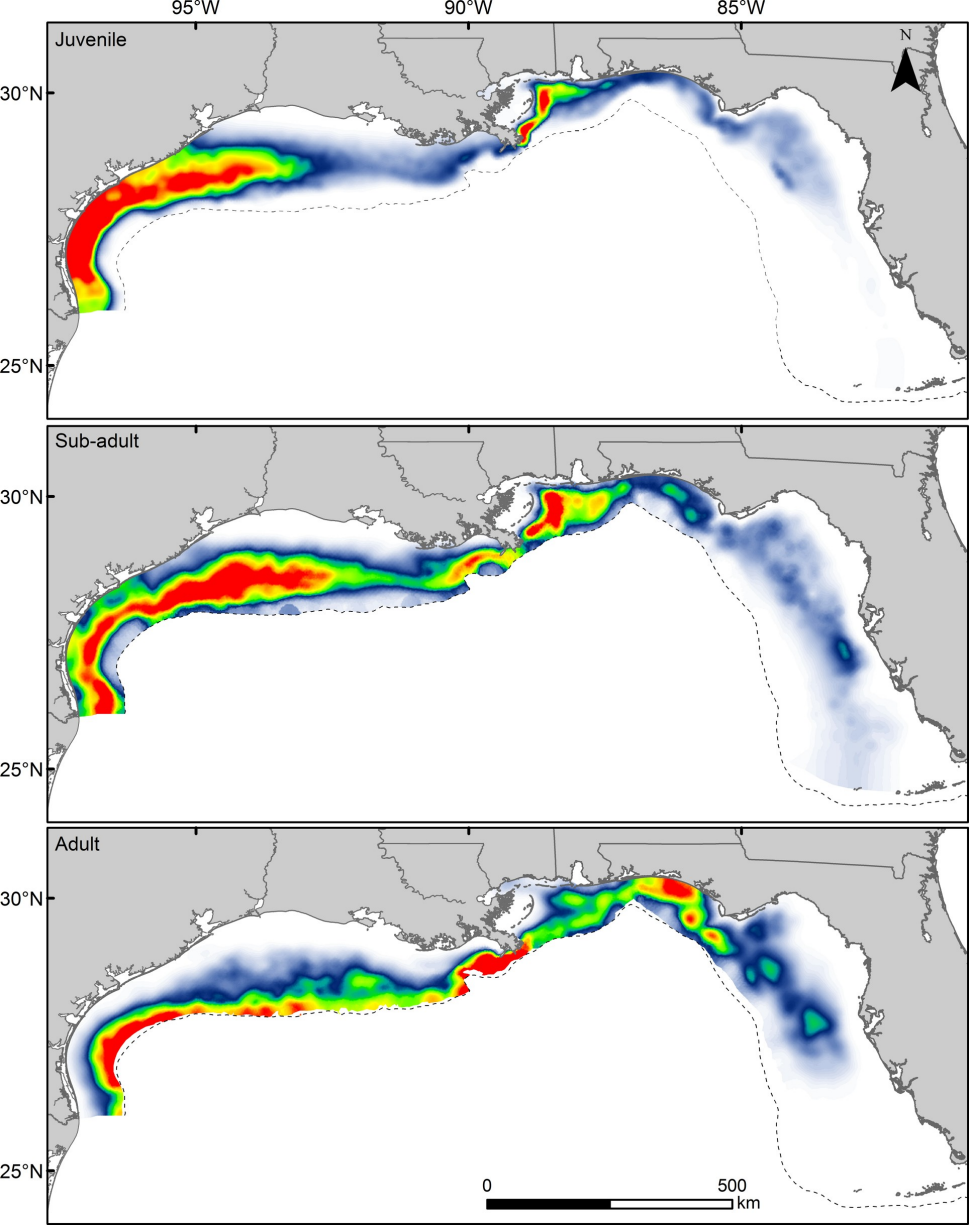
Predicted relative abundance of red snapper in shelf waters (< 150 m) of the U.S. GoM. Spatial distributions of predicted relative abundance of juvenile (age-0), sub-adult (age-1-2), and adult (age-2+) red snapper over unconsolidated substrates of the U.S. GoM based on final generalized additive models for each life stage and mean environmental conditions during the fall season (September-October). Relative abundance reflects predicted catch per trawl tow, and the scale for each panel differs: juvenile (0–56), sub-adult (0–13), and adult (0–3). Dashed line represents 150-m isobath.

Final models were used to predict the spatial distribution of red snapper habitat in the four coastal state groups (Texas, Louisiana, Mississippi/ Alabama, and Florida) for each age class (Fig 6). Overall, predicted red snapper abundance (all age classes) was higher in the western GoM relative to the eastern GoM; however, the magnitude of this discrepancy decreased with each life stage as red snapper relative abundance expanded eastward and farther offshore (Fig 6). Areal coverage of PHQ habitat varied across the four state regions during each life stage (Table 2). These regional differences were most pronounced during the juvenile stage, with over 70% (by area) of the PHQ habitat for juvenile red snapper in the GoM occurring on the Texas shelf. Similarly, a smaller area of high abundance on the Louisiana/Mississippi/Alabama shelf accounted for over 75% of the PHQ juvenile habitat in the eastern GoM (Table 2). The proportion of juvenile red snapper habitat in shelf waters of Texas and Mississippi/Alabama was greater than would be expected based on shelf area. In contrast, the proportion of juvenile red snapper habitat in Florida was much lower than would be expected based on shelf area (50% of the GoM). The percent of PHQ red snapper habitat in both Louisiana and Florida increased dramatically between the juvenile and adult stages (Table 2). For sub-adult red snapper, nearly 80% of the PHQ habitat occurred on the Texas (46.9%) and Louisiana (33.0%) shelf; however, relative to available shelf area, the proportion of high-quality habitat was highest off Texas and Mississippi/Alabama, and was lowest off Florida. Predicted adult red snapper abundance was more evenly distributed across the GoM; however, 69% of PHQ habitat for adult red snapper still occurred off Texas and Louisiana. In the western GoM, PHQ adult habitat was evenly split between Texas and Louisiana. Florida accounted for approximately 60% of the PHQ habitat of adult red snapper in the eastern GoM; however, an area that represents less than 13% of the eastern GoM shelf (Alabama, Mississippi, and Louisiana) accounted for 40% of the PHQ habitat in the eastern GoM.

**Table 2:** Relative contribution of predicted high quality habitat for red snapper in the U.S. GoM by region.

	State	% Shelf	% PHQ Habitat			PHQ Ratio		
			Juv	Sub	Adult	Juv	Sub	Adult
Overall	MS/AL	3.6	6.3	7.0	8.4	1.8	1.9	2.4
	Florida	51.3	3.5	13.2	22.1	0.1	0.3	0.4
	Louisiana	24.7	18.1	33.0	37.9	0.7	1.3	1.5
	Texas	20.4	72.1	46.8	31.6	3.5	2.3	1.5
East	MS/AL	6.1	41.1	27.1	22.8	6.8	4.5	3.8
	Florida	87.3	22.9	51.1	59.9	0.3	0.6	0.7
	Louisiana	6.6	36.0	21.8	17.3	5.4	3.3	2.6
West	Louisiana	50.4	14.9	36.9	50.0	0.3	0.7	1.0
	Texas	49.6	85.1	63.1	50.0	1.7	1.3	1.0

Regions include Mississippi/Alabama (MS/AL), Florida, Louisiana, and Texas. Metrics are shown for each region in relation to the overall, eastern (East), and western (West) U.S. GoM. % Shelf is the percent of the shelf that falls within a region. % PHQ Habitat is the percent of the total predicted high quality (PHQ) habitat for each red snapper age class within a region. PHQ Ratio is an index of relative habitat suitability expressed as the ratio of % PHQ Habitat to % Shelf. Age classes include juvenile (Juv; age-0), sub-adult (Sub; age-1-2), and adult (age-2+).

## Discussion

Depth is an important driver of reef fish community structure [41, 42] and was among the most influential variables of red snapper relative abundance across all age classes and regions. Many tropical reef fish species (e.g. snappers, groupers, grunts) undergo ontogenetic migrations from nearshore nurseries (inner shelf) to offshore spawning areas (outer shelf) [2]. Similarly, juvenile red snapper inhabit shallow water habitats, albeit these nurseries occur over sand/mud/shell substrates on the inner shelf [23, 43] as opposed to seagrass and mangrove habitats commonly used by snappers in tropical nurseries. These low-relief habitats are hypothesized to provide structure without the high densities of predators common at larger offshore reef habitats [44], suggesting shallow habitats may reduce predation and increase survival during vulnerable early life stages until individuals attain sufficient size to shift to larger structure in deeper water on the mid to outer continental shelf [45]. The observed progression to deeper benthic habitat with age is similar to dispersal patterns described for other large reef fish in subtropical systems (e.g. gag grouper; *Mycteroperca microlepis*) [46], suggesting that cross-shelf shifts during ontogeny are not unique to red snapper. Although it is also possible that observed shifts in spatial distribution and abundance may be due to differential survival due to higher fishing mortality in shallower nearshore waters [47], movement across the shelf during ontogeny is well documented in red snapper and observed depth patterns in both regions are consistent with known shifts with age to habitats of increasing complexity. Interestingly, we found that adult red snapper in the eastern GoM (where fishing pressure is greater) were associated with shallower depths (20–60 m) than those in the western GoM, where adults were most abundant at the shelf edge (100–150 m), suggesting the inshore-offshore shift with age may be less pronounced in the eastern GoM. This notion is consistent with recent findings by Powers et al. [48] off the Alabama coast and may reflect the differences in the spatial distribution of reef habitat (both artificial and natural) between the eastern and western GoM, as the majority of artificial structures in the eastern GoM are located in relatively high densities over the mid shelf in 20–60 m of water off Mississippi, Alabama, and north Florida, and age-2+ red snapper are often abundant on or near these structures [26, 49, 50]. In contrast, artificial structures in the western GoM are more widely dispersed across the shelf, and natural reefs are primarily located in deeper water (100–150 m) near the shelf edge where numerous natural banks hold high biomass of large reef fish, including red snapper [50, 51].

Physicochemical properties of seawater such as temperature, salinity, and/or dissolved oxygen concentration can influence growth, fitness, and/or survival of marine organisms [52, 53, 54]. Thus, spatial and temporal variability in these factors can play an important role in determining habitat quality and regulating the distributions of marine fishes [36, 55]. Dissolved oxygen concentration was among the most important predictor variables for juvenile red snapper, which is not surprising given that hypoxia typically reduces nursery habitat quality and negatively impacts recruitment [56, 57]. The finding that juvenile red snapper abundance was negatively associated with low dissolved oxygen concentration is consistent with previous studies that have implicated hypoxia as a source of recruitment variability for this species [58–60]. In the western GoM, a large hypoxic zone forms off the Louisiana coast during the summer and fall, which may limit the recruitment of red snapper over the inner shelf and explain the observed decrease in juvenile abundance from west to east (Texas to Louisiana) [61]. Juvenile red snapper were also more abundant in areas with higher bottom temperatures, suggesting a potential thermal preference by juveniles that may maximize growth [62]. Alternatively, this pattern could simply reflect benthic habitat preference for the inner shelf where bottom temperatures are warmer during summer and fall relative to outer shelf habitats that receive cooler upwelled water from the shelf break that intrudes onto the shelf [63].

The importance of habitat complexity to reef fishes is well documented, and ontogenetic habitat shifts are often attributed to the need for increased structural complexity to maximize growth and minimize mortality [2, 44]. Our finding that associations with hard bottom habitat increased with age corroborates previous research that suggests red snapper move from areas of relative low complexity (sand, shell) to areas of higher complexity (natural reef banks) as they mature [26]. Although sampling occurred over unconsolidated substrates, the relative abundance of adult red snapper increased within 10–20 km of natural hard bottom habitat, which might be expected given the high abundance of adult red snapper at natural banks in the GoM [50, 51]. Adult red snapper in the GoM are thought to become less reef dependent and increasingly common over mud/sand bottom with age [26]; however, this shift is not observed in other regions of the western Atlantic, which has been attributed to lower population density and/or greater availability of hard bottom habitat [64]. While it is clear that adult red snapper are common on unconsolidated substrates in the GoM, our results suggest that abundance over these areas is still likely influenced by proximity to reef structure.

Artificial structures harbor high densities of reef fishes and have been deployed in shelf waters worldwide, yet relatively little is known about how these structures influence the distribution of marine organisms over large spatial scales. While areal coverage of artificial structure relative to natural hard bottom in the GoM is minimal, red snapper densities are often higher at artificial structures [51], and a recent study estimated that a significant proportion (~ 13%) of red snapper in the GoM are found at these habitats [50]. In the current study, we found the influence of artificial structure on red snapper abundance over unconsolidated bottom was both age- and region-specific. The finding that juvenile red snapper were negatively associated with proximity to artificial structure is not surprising given the high densities of adult red snapper and other predatory reef fish associated with these habitats [49, 65], and supports the notion that red snapper generally do not recruit to artificial structures until age-1+ [24, 26]. At sub-adult and adult life stages, region-specific differences in the composition and spatial configuration of the seascapes associated with artificial structures between the eastern and western GoM may influence distribution and abundance of red snapper on surrounding unconsolidated substrates. Relative abundance of adult red snapper over unconsolidated substrate was positively associated with artificial structures in the eastern GoM, which is characterized by several large artificial reef zones from Louisiana to Northwest Florida that contain high densities of prefabricated concrete structures (i.e. reef balls, pyramids) [49, 50]. The large footprints of these reef zones and high likelihood of encountering another structure in close proximity may facilitate movement of red snapper among structures [66, 67] and reduce predation risk associated with straying. This type of movement and exchange may increase abundance over unconsolidated substrates surrounding artificial structures. In contrast, the majority of artificial structures in the western GoM are oil and gas platforms that provide mostly vertical relief, and are relatively isolated compared to lower profile structures that are more common in the eastern GoM. Large predators (i.e. sharks, barracudas) are relatively common at oil and gas platforms [65], which may increase the associated risk of straying from the structure [5]. Close association of fish to the platform structures would limit vulnerability to trawling gear, and could explain why artificial structures had little influence on adult red snapper abundance over surrounding unconsolidated bottom in the western GoM. Alternatively, it is also possible that these trends simply reflect density dependent processes related to differences in population size [64], and larger red snapper in the western GoM disproportionately use non-structured habitat away from reefs relative to the smaller eastern subpopulation.

Distribution and abundance of red snapper across unconsolidated bottom of the GoM was strongly influenced by geographic variables such as longitude and latitude. Spatial patterns in juvenile abundance likely reflect basin-scale patterns of recruitment that are influenced by both larval transport and the distribution of suitable habitat. While red snapper abundance was not correlated with benthic substrate at smaller spatial scales, likely due to limited resolution of available data layers [50], geographic trends indicated higher juvenile abundance in regions (south Texas and Louisiana/Mississippi shelves) characterized by broad areas of mud and scattered low-relief habitat preferred by this species during early life [23, 24]. In contrast, low juvenile abundance was associated with areas characterized by unfavorable physical conditions (hypoxic zone in western GoM) or reduced larval supply (eastern GoM). Larval catch data and particle transport models suggest that larval supply likely varies across the GoM, and may limit recruitment to areas such as the West Florida Shelf where adult biomass is relatively low and supply from other regions is limited [31, 68–71]. In addition, the West Florida Shelf also represents a transition from subtropical to tropical ecoregions [72], and an increase in mangrove-seagrass nurseries and nearshore reef systems preferred by tropical snappers and groupers may increase competition and limit nursery habitat quality. Alternatively, observed spatial patterns could reflect differential catchability of juvenile red snapper in trawls on the West Florida Shelf due to the greater amount of natural hard bottom in the eastern GoM. However, this seems unlikely as trawl surveys were not conducted in known reef areas, the gear was effective at capturing larger red snapper in the region which are theoretically less vulnerable to trawling gear, and our findings are in accord with recent stock assessments that indicate juvenile recruitment to the West Florida Shelf is low [32].

Maintaining connectivity between juvenile and adult habitats is important to sustaining reef fish populations [73]. Genetic evidence indicates that red snapper in the GoM follow a metapopulation structure, with limited long-term gene flow among semi-isolated assemblages that are demographically independent over short temporal scales [74]. Specifically, movement (passive or active) across the Mississippi River plume appears to be limited [67, 69], and recent stock assessments indicate that the eastern and western subpopulations are recovering at different trajectories [29, 75]. In both the eastern and western GoM, we observed shifts in relative abundance of red snapper over unconsolidated substrate from west to east with age, and differences in these patterns may have important implications for population connectivity. A clear cross-shelf shift in abundance occurs with ontogeny in the western GoM, while in the eastern GoM red snapper expand eastward with age from areas of high juvenile abundance off the Mississippi/Alabama and Louisiana shelves. Stronger inshore-offshore movement with age in the western GoM may be a function of the spatial distribution of nursery habitat relative to hard bottom habitat which is located near the shelf edge in the western GoM, while both hard bottom and artificial structures are prevalent on the mid shelf in the eastern GoM and generally east of the juvenile habitat [76]. While observed shifts in abundance from west to east with age could also reflect differential survival across regions and/or depth zones (see Frank et al. [47]), this pattern is consistent with long-term tagging studies that have documented net eastwardly movement of red snapper from artificial reef sites off Alabama and western Florida [67, 77], suggesting that nursery grounds in the north from Louisiana to the Florida Panhandle may be an important source of recruits to the West Florida Shelf (shelf waters south and east of Cape San Blas, Florida). Given the aforementioned differences in the recovery of the two subpopulations, regional differences in patterns of dispersal and connectivity may have important implications for rebuilding efforts in both regions.

While red snapper occur throughout the GoM, regional differences in the availability of suitable habitat may have important implications for the management of the species. Our models indicated that the vast majority of predicted high quality (PHQ) habitat for juvenile red snapper was concentrated on the inner shelf off of Texas and a smaller area east of the Mississippi River Delta (Louisiana and Mississippi/Alabama). Not surprisingly, these areas are similar in that both are characterized by relatively high larval supply [68, 69] and the presence of mud/shell ridge habitats that provide an ideal level of complexity for red snapper settlement [23, 24]. Although the western GoM contains far more PHQ habitat, both areas appear to represent critical nursery or production zones with the PHQ habitat east of the Mississippi River Delta possibly serving as a critical source of juvenile recruits to the eastern subpopulation. Interestingly, the PHQ habitat of juvenile red snapper in the eastern GoM is proximal to substantial artificial reef habitat on the Mississippi/Alabama shelf, and therefore may contribute to the locally high abundance of sub-adult and adult red snapper in this region [50]. Historically, Northwest Florida was the center of the early red snapper fishery, and recent stock assessments suggest that populations off Florida remain depleted relative to historical abundance [32]. Despite large areas of natural hard bottom, Florida accounted for less PHQ habitat at each age class than would be expected based on shelf area (51%), and similar to previous studies [78, 79], we found the majority of habitat was concentrated in the northwestern portion of the state, in closer proximity to the aforementioned PHQ nursery habitat east of the Mississippi River. While the mechanism (e.g., larval supply, benthic structure, historical depletion, predation) limiting the areal coverage of high-quality habitat for juvenile red snapper in Florida warrants future study, the relative lack of juvenile red snapper on the West Florida Shelf is concerning considering the high fishing pressure in the region [80–82]. Despite our finding that the western GoM contains twice as much adult habitat, the recent stock assessment indicated that recreational fishing pressure in the eastern GoM was three-fold greater than the western GoM [32]. In addition, Florida accounted for the highest proportion of red snapper catch among GoM states in both recreational and commercial sectors from 2008–2014. The lack of PHQ nursery habitat (i.e., production) for red snapper in Florida indicates a potential reliance on juveniles produced in other areas with similarly high fishing pressure (e.g. Mississippi/Alabama). This may hinder the recovery of the eastern subpopulation, making populations in Florida more vulnerable to overfishing and fishing activities in surrounding states compared to other regions of the GoM. It is also possible that the lack of PHQ habitat for juvenile red snapper on the West Florida Shelf reflects the relatively depleted state of the eastern subpopulation during much of the study period [32]. If so, areas of lower quality juvenile habitat may become occupied, given greater adult biomass and/or increased larval supply from an increasing population (i.e. MacCall’s basin model) [83].

Previous studies have used fisheries independent data sets from SEAMAP surveys and similar environmental data layers to model spatial distributions of marine fishes and invertebrates in the GoM [79, 84–86]. While the use of these data to model spatial distributions and habitat associations of marine organisms is promising, there remain limitations that should be considered when interpreting results presented here. First, while data layers describing the spatial distribution of bottom type represent the best available data, these data are relatively coarse in resolution and rely on spatial interpolation. Second, SEAMAP sampling protocols avoid artificial and natural reef habitat, and although sampling stations during the study period were often located in close proximity (< 1 km) to one or both habitat types, samples were not taken directly from either artificial or natural reef habitats that are known to be important habitat for sub-adult and adult red snapper. While these limitations may explain why habitat variables had less influence on sub-adult and adult red snapper abundance relative to other environmental variables in some of our models, particularly in the western GoM, it is important to reiterate that fish-habitat relationships described here primarily apply to red snapper found over unconsolidated substrates. Nevertheless, GoM-scale spatial patterns of sub-adult and adult red snapper abundance over unconsolidated substrate described here are in general agreement with spatial predictions based on comprehensive reef sampling described by Kaurnauskas et al. [50], suggesting that spatial patterns of red snapper abundance over unconsolidated substrates are reflective of regional patterns of red snapper distribution. The inclusion of latitude and longitude as explanatory variables likely helped account for geographic variability in the distribution of benthic habitat and uncertainty in habitat data layers, improving predictions. Lastly, these limitations highlight the challenges in conducting standardized fisheries-independent sampling across both trawlable and untrawlable habitat in the GoM. Large-scale efforts are currently underway to address these issues by employing multiple gear types over both unconsolidated and consolidated substrates, with the goal of providing a better understanding of the distribution and abundance of adult reef fish in the GoM.

Our results reveal clear habitat shifts with age for red snapper and demonstrate that fish-habitat relationships vary regionally within an age/size structured population. Moreover, observed differences in patterns of connectivity across the GoM may drive regional population dynamics and future species management. Adult stocks in regions that rely on local production (western GoM) will likely respond differently to anthropogenic factors (fishing pressure, habitat loss) than stocks in regions (e.g., West Florida Shelf) that are more reliant on juvenile production from surrounding areas. In the latter scenario, changes in management or fishing pressure in regions of high juvenile production will likely have major impacts on adult stocks in surrounding regions with negligible juvenile production. While our findings also begin to address how PHQ habitat varies temporally, additional research is needed to better understand how dynamic processes such as spawning, larval transport, and survival influence spatial and temporal variability in predicted habitat quality. Successful recovery of both subpopulations will require management strategies that carefully consider spatially explicit information on distribution that documents natural variability in habitat quality.

## Supporting information

S1 Fig Map showing administrative boundaries dividing shelf waters of the U.S. Gulf of Mexico among four coastal state groups. State groupings include Texas (TX), Louisiana (LA), Mississippi/Alabama (MS/AL), and Florida (FL). For the purposes of this study shelf waters of Mississippi and Alabama were combined. Administrative boundaries were defined by the U.S. Bureau of Ocean Energy Management (BOEM).
(TIF)

S2 Fig Distribution of predicted high quality (PHQ) habitat for juvenile (age-0), sub-adult (age 1–2), and adult (age 2+) red snapper in the U.S. Gulf of Mexico. PHQ habitat was defined as areas constituting the upper 95% of predicted abundance at each life stage. Predictions were based on final generalized additive models for each life stage and mean environmental conditions during the fall season (September-October). Dashed line represents 150-m isobaths.
(TIF)

S1 Appendix Maps showing the distribution of relative standard error (SE, Fig A), predicted lower bounds (fitted values–SE, Fig B), and predicted upper bounds (fitted values + SE, Fig C) for red snapper relative abundance estimates by age class during fall in the U.S. Gulf of Mexico based on final GAMs. As expected, error estimates were relatively higher in areas with higher relative abundance. Maps showing the distribution of lower and upper bounds demonstrate very little deviation from the relative distribution of red snapper abundance based on fitted values shown in Fig 6.
(PDF)
