# Supplementary figures and images for: Cross-shelf habitat shifts by red snapper (Lutjanus campechanus) in the Gulf of Mexico
Source: PLoS One. 2019 Mar 14;14(3):e0213506. doi: 10.1371/journal.pone.0213506 (PMC6417787; doi:10.1371/journal.pone.0213506)

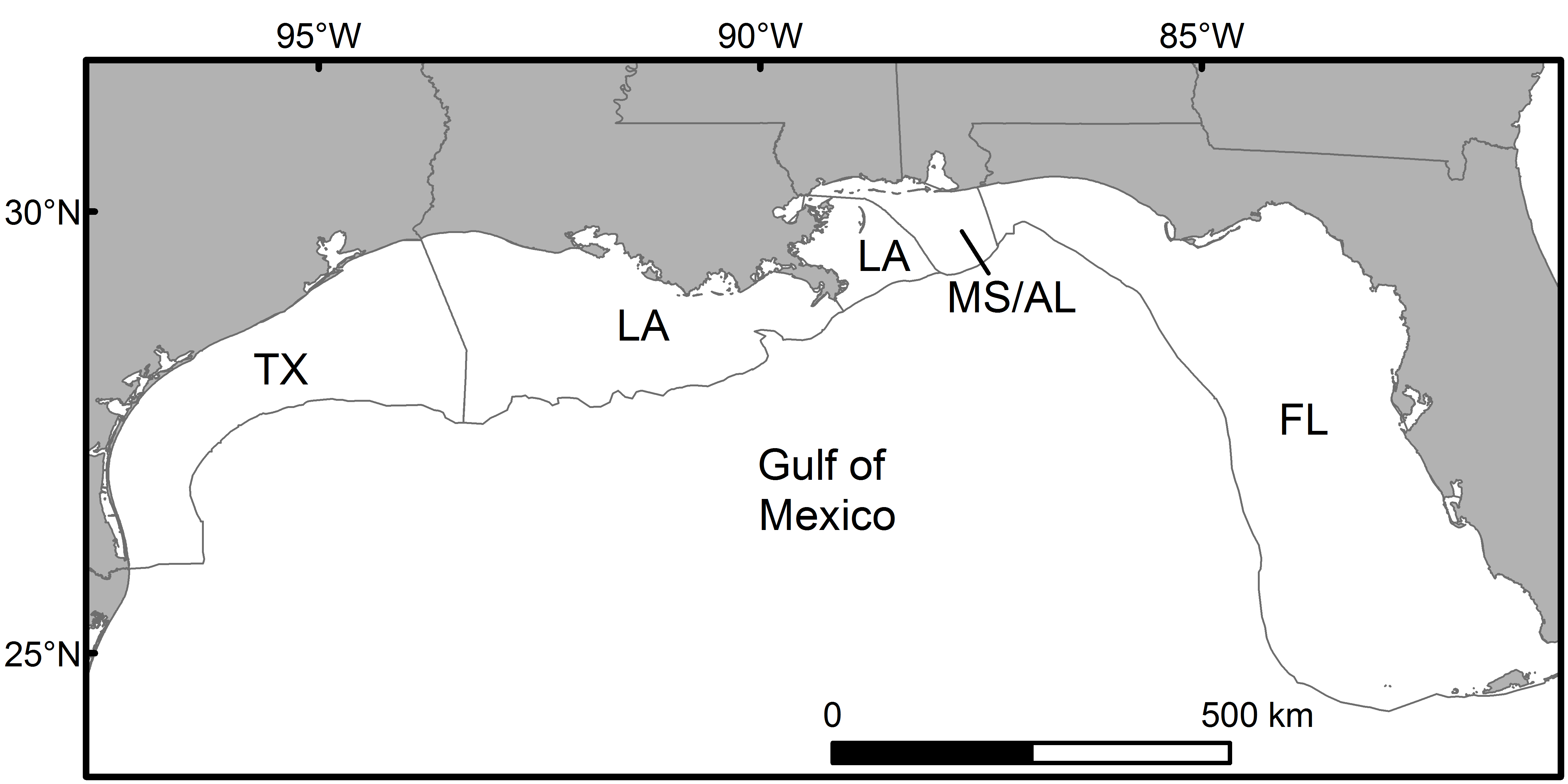

Supplement: S1 Fig — State groupings include Texas (TX), Louisiana (LA), Mississippi/Alabama (MS/AL), and Florida (FL). For the purposes of this study shelf waters of Mississippi and Alabama were combined. Administrative boundaries were defined by the U.S. Bureau of Ocean Energy Management (BOEM). (TIF) [file pone.0213506.s001.tif]

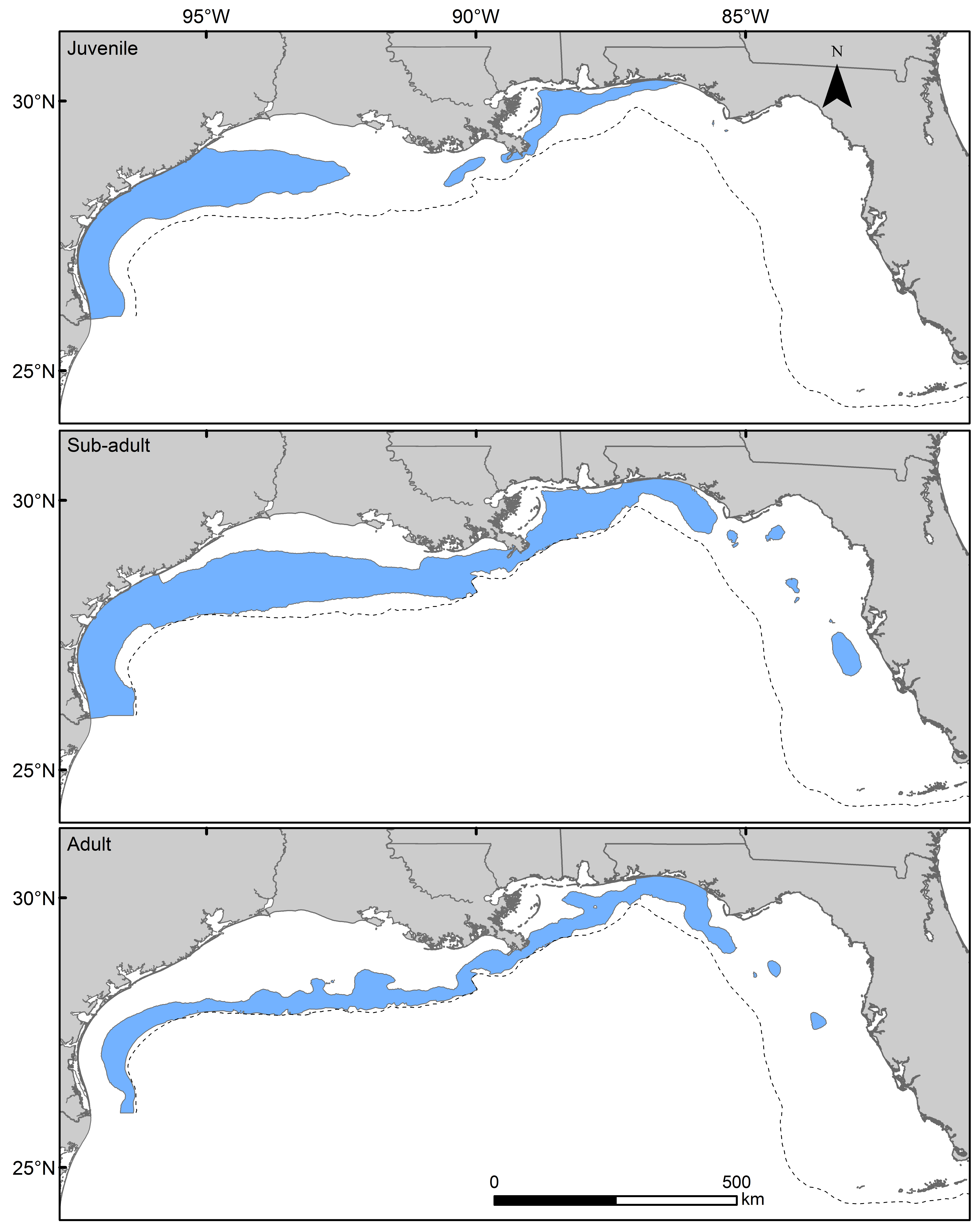

Supplement: S2 Fig — PHQ habitat was defined as areas constituting the upper 95% of predicted abundance at each life stage. Predictions were based on final generalized additive models for each life stage and mean environmental conditions during the fall season (September-October). Dashed line represents 150-m isobaths. (TIF) [file pone.0213506.s002.tif]
