# Supplementary material for: Cross-shelf habitat shifts by red snapper (Lutjanus campechanus) in the Gulf of Mexico
Source: PLoS One. 2019 Mar 14;14(3):e0213506. doi: 10.1371/journal.pone.0213506 (PMC6417787; doi:10.1371/journal.pone.0213506)

S1 Appendix

Fig A. Relative distribution of standard error (SE) for predicted red snapper relative abundance during the fall based on final GAMs.

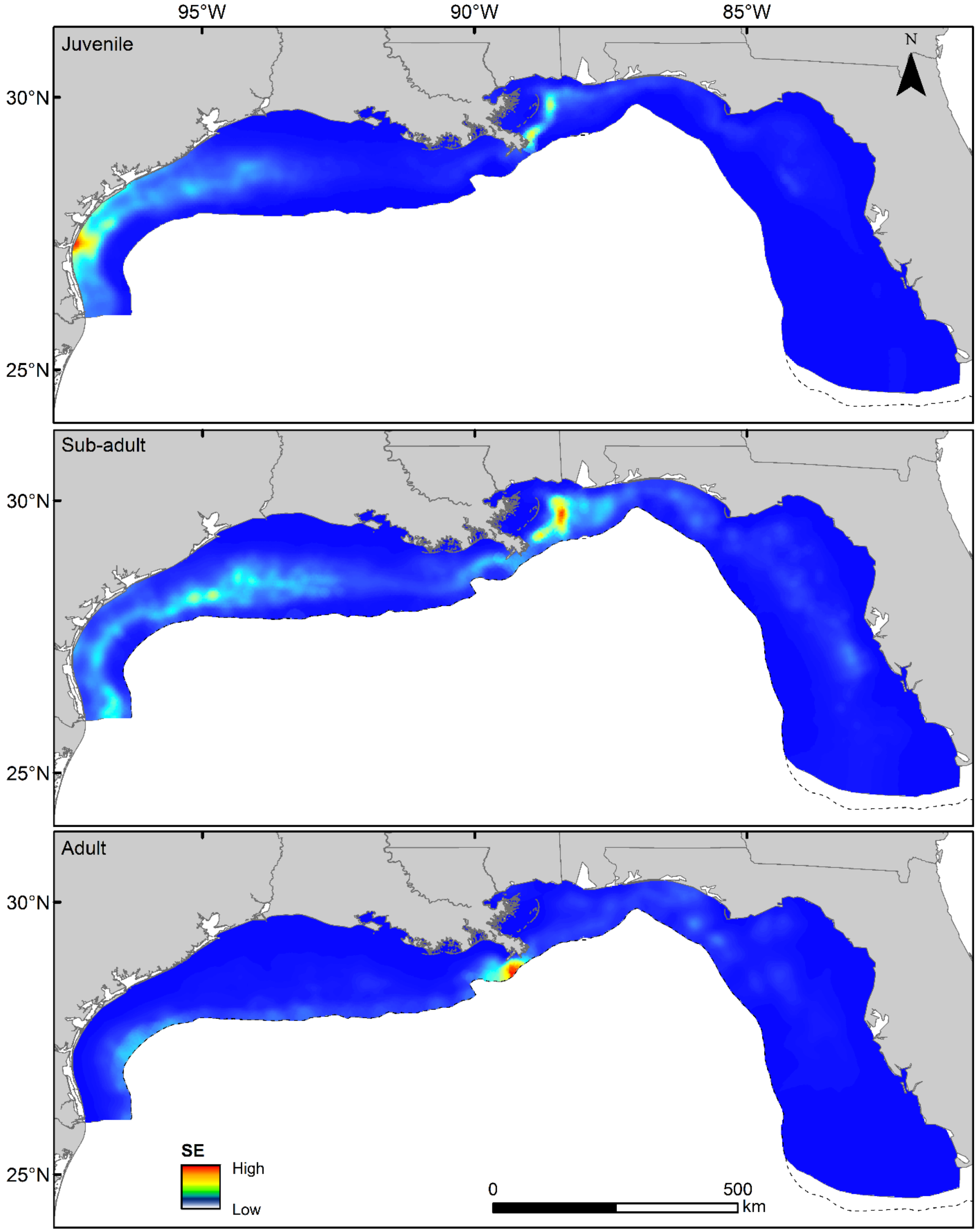

Fig B. Plots showing the distribution of the lower bounds (fitted value – SE) of red snapper predicted relative abundance by age class.

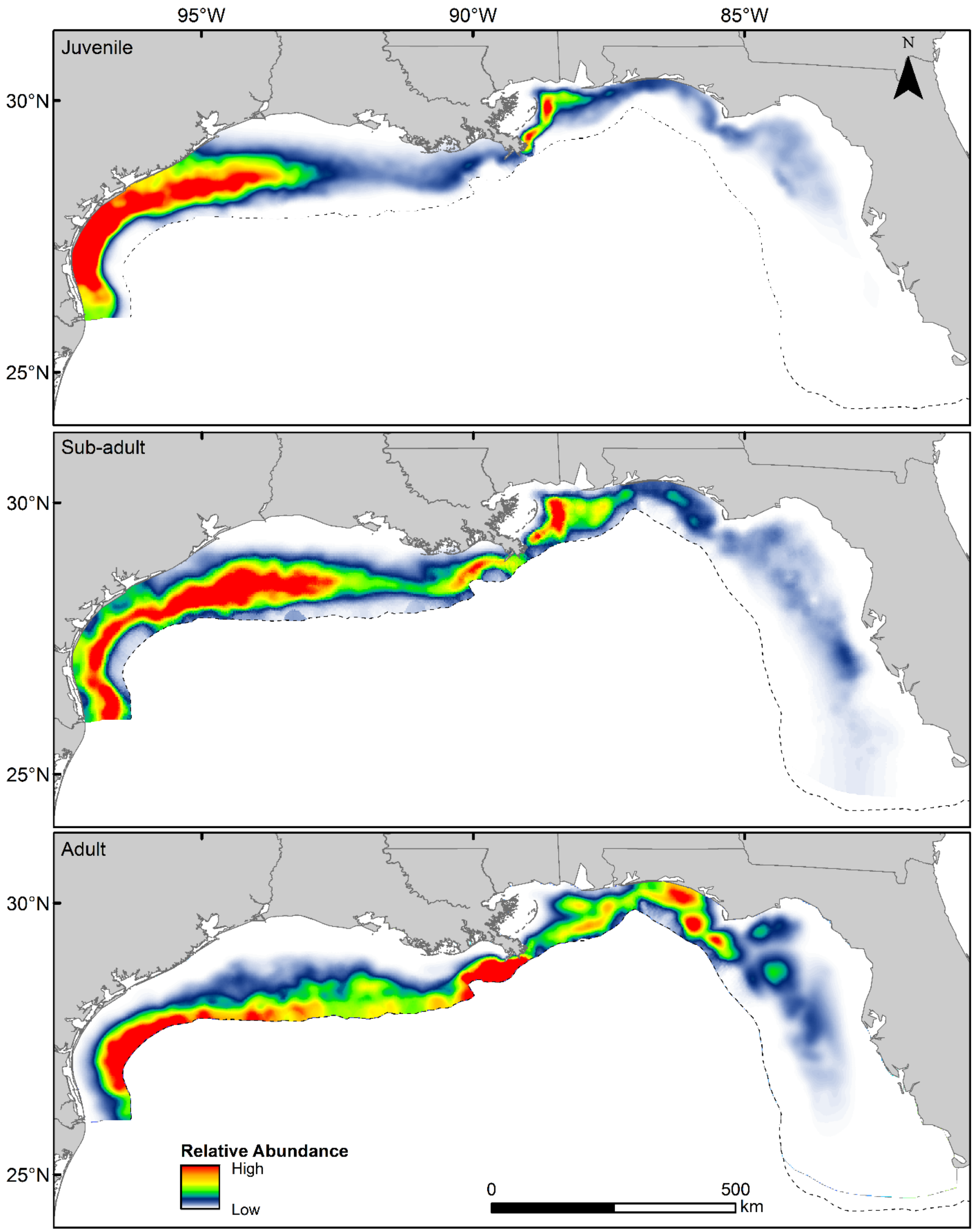

Fig C. Plots showing the distribution of the upper bounds (fitted value + SE) of red snapper predicted relative abundance by age class.

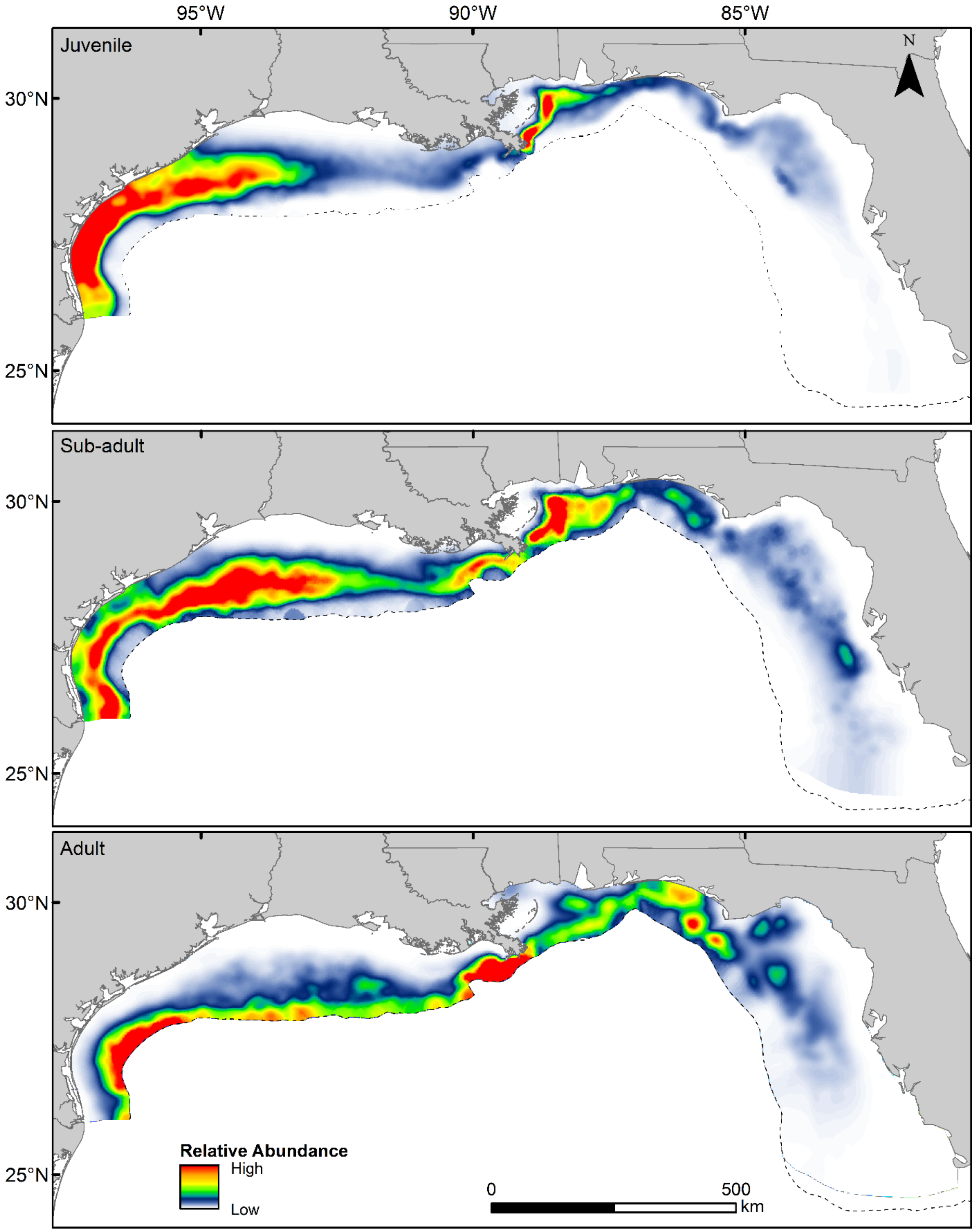

Supplement: S1 Appendix — As expected, error estimates were relatively higher in areas with higher relative abundance. Maps showing the distribution of lower and upper bounds demonstrate very little deviation from the relative distribution of red snapper abundance based on fitted values shown in Fig 6. (PDF) [file pone.0213506.s003.pdf]
